# Supplementary material for: Disentangling the roles of different vector species during a malaria resurgence in Eastern Uganda
Source: PLOS Glob Public Health. 2025 Dec 11;5(12):e0004436. doi: 10.1371/journal.pgph.0004436 (PMC12697997; doi:10.1371/journal.pgph.0004436)
Supplement: S1 Text — X’s in columns indicate whether a model contained a spatial smooth or a household random effect. Models are compared using Akaike information criterion (AIC) and percentage of deviance explained. The models selected for further analysis are bolded. (DOCX) [file pgph.0004436.s001.docx]

**S1 Text. Comparison of candidate entomological generalized additive models by geographic zone**.

X’s in columns indicate whether a model contained a spatial smooth or a household random effect. Models are compared using Akaike information criterion (AIC) and percentage of deviance explained. The models selected for further analysis are bolded.

**Table A. Comparison of candidate *An. gambiae* vector count generalized additive models by geographic zone.**

| An. gambiae counts | |  |  |  |
| --- | --- | --- | --- | --- |
| Zone | **HHID RE** | **Spatial** | **AIC** | **% Deviance explained** |
| Busia |  |  | 10347.218 | 39.7 |
|  | **x** |  | **9480.547** | **62.5** |
|  |  | x | 9518.456 | 61.3 |
| Tororo, near | |  | 17512.39 | 32 |
|  | **x** |  | **15908.41** | **56.7** |
|  |  | x | 15911.16 | 56.6 |
| Tororo, away | |  | 8861.704 | 11.4 |
|  | **x** |  | **8106.63** | **35.1** |
|  |  | x | 8108.343 | 35.1 |

**Table B. Comparison of candidate *An. funestus* vector count generalized additive models by geographic zone.**

| An. funestus counts | |  |  |  |
| --- | --- | --- | --- | --- |
| Zone | **HHID RE** | **Spatial** | **AIC** | **% Deviance explained** |
| Busia |  |  | 6241.449 | 22.3 |
|  | **x** |  | **5636.26** | **48.7** |
|  |  | x | 5663.009 | 47.5 |
| Tororo, near | |  | 14317.54 | 19.5 |
|  | **x** |  | **13276.02** | **41.6** |
|  |  | x | 13279.96 | 41.5 |
| Tororo, away | |  | 6271.377 | 24.8 |
|  | **x** |  | **5842.309** | **41.6** |
|  |  | x | 5841.452 | 41.3 |

**Table C. Comparison of candidate *An. gambiae* sporozoite rate generalized additive models by geographic zone.**

| An. gambiae SR |  |  |  |
| --- | --- | --- | --- |
| Zone | **HHID RE** | **AIC** | **% Deviance explained** |
| Busia |  | 839.7793 | 8.72 |
|  | **x** | **835.2698** | **11.3** |
| Tororo, near | | 948.259 | 10.7 |
|  | **x** | **948.2594** | **10.7** |
| Tororo, away | | 304.4904 | 7.13 |
|  | **x** | **294.5583** | **19.7** |

**Table D. Comparison of candidate *An. funestus* sporozoite rate generalized additive models by geographic zone.**

| An. funestus SR |  |  |  |
| --- | --- | --- | --- |
| Zone | **HHID RE** | **AIC** | **% Deviance explained** |
| Busia |  | 382.826 | 0.0662 |
|  | **x** | **382.826** | **0.0663** |
| Tororo, near | | 800.4146 | 14.1 |
|  | **x** | **789.3133** | **19.5** |
| Tororo, away | | 302.3484 | 14.3 |
|  | **x** | **302.3494** | **14.3** |
