# Supplementary figures and images for: Disentangling the roles of different vector species during a malaria resurgence in Eastern Uganda
Source: PLOS Glob Public Health. 2025 Dec 11;5(12):e0004436. doi: 10.1371/journal.pgph.0004436 (PMC12697997; doi:10.1371/journal.pgph.0004436)

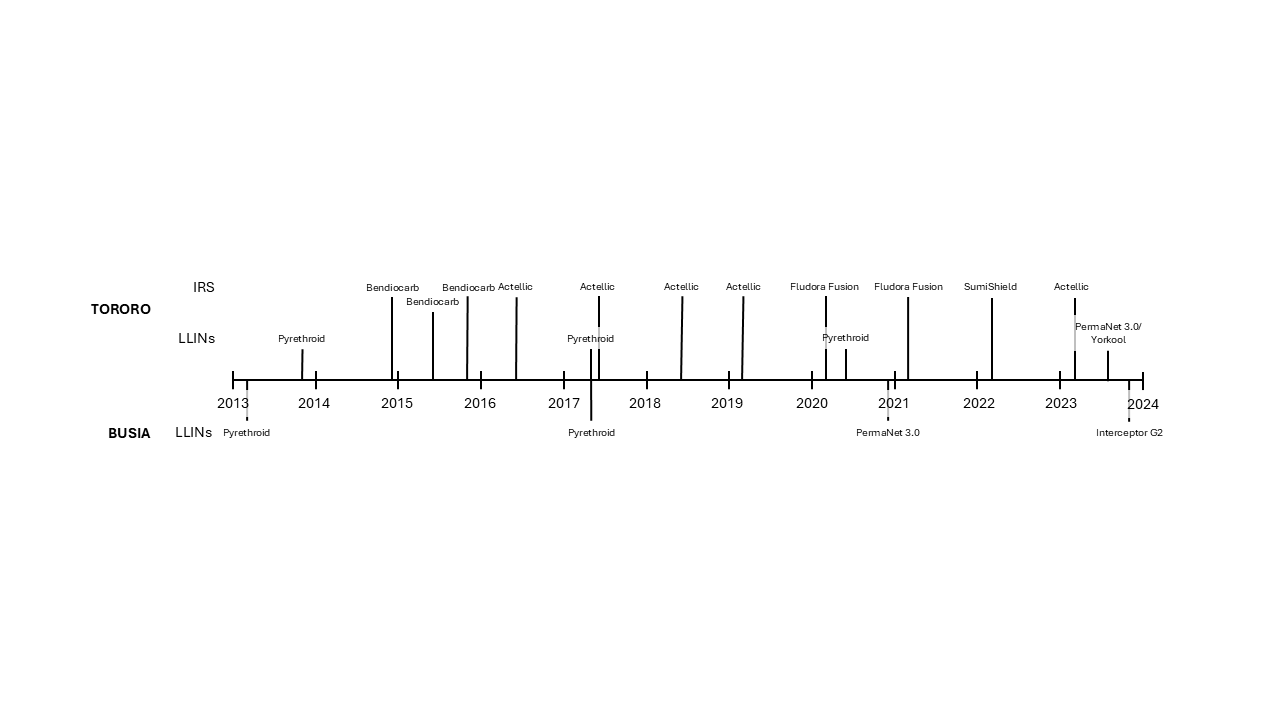

Supplement: S1 Fig — Timeline of recent indoor residual spraying (IRS) and long-lasting insecticidal net (LLIN) distribution campaigns by district, including names of insecticide formulations and LLIN models. (PNG) [file pgph.0004436.s002.png]

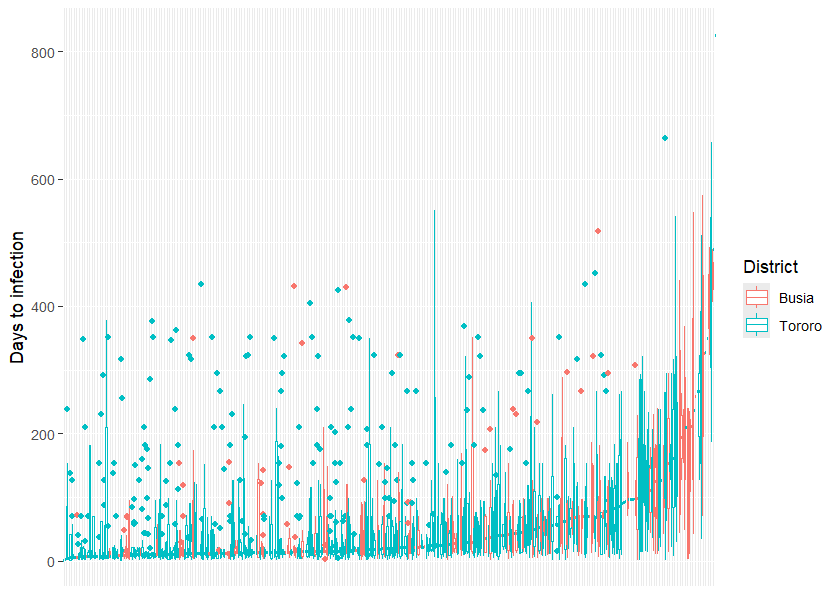

Supplement: S2 Fig — Each boxplot represents the distribution of gap times in days for an individual participant. The boxplot includes marks indicating the median and first and third quartiles, two whiskers, and outlying points, with color varying by site. (TIFF) [file pgph.0004436.s003.tiff]

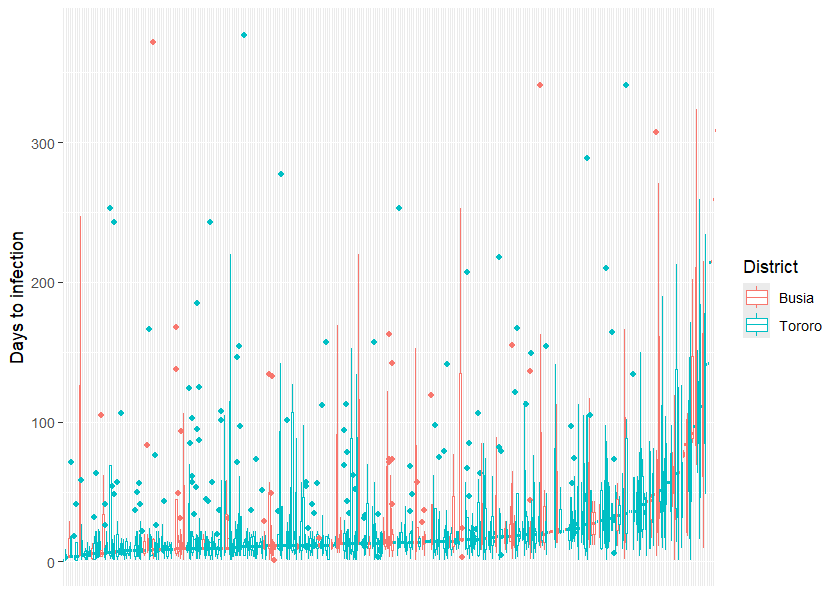

Supplement: S3 Fig — Each boxplot represents the distribution of gap times in days for an individual participant. The boxplot includes marks indicating the median and first and third quartiles, two whiskers, and outlying points, with color varying by site. (TIFF) [file pgph.0004436.s004.tiff]
