# Supplementary material for: Disentangling the roles of different vector species during a malaria resurgence in Eastern Uganda
Source: PLOS Glob Public Health. 2025 Dec 11;5(12):e0004436. doi: 10.1371/journal.pgph.0004436 (PMC12697997; doi:10.1371/journal.pgph.0004436)
Supplement: S1 Table — For the purposes of calculating infection incidence, person-time here omits all days between an incident infection and day 14 following the individual’s next malaria treatment (see Methods). (DOCX) [file pgph.0004436.s005.docx]

**S1 Table. Disregarding natural clearances: participant characteristics**

|  | Busia | Tororo, near | Tororo, away | Overall |
| --- | --- | --- | --- | --- |
| No. indiv. | 93 | 149 | 133 | 375 |
| No. HH | 23 | 38 | 31 | 92 |
| % Male | 50.5 | 53.0 | 47.4 | 50.4 |
| Median proportion of nights using LLIN (IQR) | 1 (1, 1) | 1 (1, 1) | 1 (1, 1) | 1 (1, 1) |
| Median age in years (IQR) | 5.1 (3.2, 10) | 5.3 (2.8, 8.8) | 5.3 (3, 9.4) | 5.3 (3, 9.3) |
| Median days followed (IQR) | 955 (576, 1139) | 1124 (776, 1131) | 977 (616, 979) | 978 (607, 1126) |
| Median infections per person-year (IQR) | 5.4 (2.8, 9.8) | 6.3 (3.3, 10.5) | 4.7 (3.1, 8) | 5.4 (3, 9.4) |
| Median cases malaria per person-year (IQR) | 2 (1, 3.2) | 2.5 (1.4, 3.3) | 2.1 (1.6, 3.7) | 2.1 (1.3, 3.3) |
