# Supplementary material for: Disentangling the roles of different vector species during a malaria resurgence in Eastern Uganda
Source: PLOS Glob Public Health. 2025 Dec 11;5(12):e0004436. doi: 10.1371/journal.pgph.0004436 (PMC12697997; doi:10.1371/journal.pgph.0004436)
Supplement: S2 Table — All aEIRs are log2-transformed. (DOCX) [file pgph.0004436.s006.docx]

**S2 Table. Reanalysis including interactions between time period relative to malaria resurgence and aEIR.**

All aEIRs are log_2_-transformed.

|  | Busia | Tororo | Overall |
| --- | --- | --- | --- |
| Total aEIR | | | |
| Age (years) | 1.050 (1.020,1.09) | 1.01 (0.997,1.030) | 1.020 (1.010,1.040) |
| Total aEIR | 1.280 (1.170,1.41) | 1.25 (1.160,1.350) | 1.310 (1.240,1.390) |
| During | 2.500 (1.420,4.40) | 3.70 (2.600,5.280) | 4.380 (3.280,5.840) |
| After | 1.520 (0.655,3.51) | 1.44 (0.933,2.230) | 1.980 (1.400,2.790) |
| Total aEIR:During | 0.932 (0.838,1.04) | 1.06 (0.971,1.160) | 0.959 (0.899,1.020) |
| Total aEIR:After | 1.030 (0.880,1.19) | 1.27 (1.110,1.460) | 1.060 (0.978,1.150) |
| Sp.-specific aEIRs | | | |
| Age (years) | 1.060 (1.020,1.09) | 1.01 (0.995,1.030) | 1.020 (1.000,1.040) |
| An. funestus aEIR | 1.270 (1.080,1.49) | 1.14 (1.040,1.250) | 1.190 (1.100,1.290) |
| An. gambiae aEIR | 1.120 (1.010,1.24) | 1.15 (1.060,1.250) | 1.170 (1.100,1.240) |
| During | 2.510 (1.490,4.21) | 4.00 (2.910,5.490) | 4.140 (3.200,5.360) |
| After | 2.040 (0.988,4.20) | 1.49 (1.000,2.230) | 2.080 (1.530,2.830) |
| An. funestus aEIR:During | 1.030 (0.828,1.28) | 1.14 (1.030,1.260) | 1.130 (1.040,1.230) |
| An. funestus aEIR:After | 0.867 (0.710,1.06) | 1.29 (1.110,1.490) | 1.040 (0.937,1.160) |
| An. gambiae aEIR:During | 0.889 (0.763,1.04) | 0.89 (0.810,0.978) | 0.822 (0.765,0.882) |
| An. gambiae aEIR:After | 1.060 (0.914,1.23) | 1.11 (0.936,1.300) | 0.990 (0.896,1.090) |
