# Supplementary material for: Disentangling the roles of different vector species during a malaria resurgence in Eastern Uganda
Source: PLOS Glob Public Health. 2025 Dec 11;5(12):e0004436. doi: 10.1371/journal.pgph.0004436 (PMC12697997; doi:10.1371/journal.pgph.0004436)
Supplement: S3 Table — Models fit to expected EIRs. All aEIRs are log2-transformed. (DOCX) [file pgph.0004436.s007.docx]

**S3 Table. Disregarding natural clearances: aHRs assuming a linear relationship between all covariates and the log hazard.**

Models fit to expected EIRs. All aEIRs are log_2_-transformed.

|  | Busia | Tororo | Overall |
| --- | --- | --- | --- |
| Total aEIR | | | |
| Total aEIR | 1.23 (1.170,1.30) | 1.190 (1.140,1.24) | 1.190 (1.150,1.23) |
| Age (years) | 1.05 (1.020,1.09) | 0.987 (0.971,1.00) | 0.988 (0.974,1.00) |
| During | 1.81 (1.430,2.29) | 2.450 (2.060,2.90) | 2.300 (2.000,2.65) |
| After | 1.69 (1.260,2.27) | 1.420 (1.110,1.81) | 1.520 (1.250,1.84) |
| Sp.-specific aEIRs | | | |
| An. funestus aEIR | 1.08 (0.987,1.19) | 1.150 (1.100,1.20) | 1.290 (1.250,1.34) |
| An. gambiae aEIR | 1.12 (1.050,1.20) | 1.050 (1.000,1.10) | 1.040 (1.010,1.08) |
| Age (years) | 1.00 (0.974,1.03) | 0.987 (0.971,1.00) | 1.020 (1.000,1.04) |
| During | 1.67 (1.280,2.16) | 2.120 (1.760,2.55) | 2.970 (2.620,3.37) |
| After | 1.73 (1.220,2.44) | 1.250 (0.976,1.59) | 1.870 (1.570,2.22) |
