# Supplementary material for: Disentangling the roles of different vector species during a malaria resurgence in Eastern Uganda
Source: PLOS Glob Public Health. 2025 Dec 11;5(12):e0004436. doi: 10.1371/journal.pgph.0004436 (PMC12697997; doi:10.1371/journal.pgph.0004436)
Supplement: S4 Table — Models fit to expected EIRs. All aEIRs are log2-transformed. (DOCX) [file pgph.0004436.s008.docx]

**S4 Table. 28-day lagged aEIR: aHRs assuming a linear relationship between all covariates and the log hazard.**

Models fit to expected EIRs. All aEIRs are log_2_-transformed.

|  | Busia | Tororo | Overall |
| --- | --- | --- | --- |
| Total aEIR | | | |
| Total aEIR | 1.22 (1.150,1.28) | 1.30 (1.250,1.36) | 1.26 (1.22,1.30) |
| Age (years) | 1.05 (1.020,1.08) | 1.02 (1.000,1.03) | 1.02 (1.01,1.04) |
| During | 1.69 (1.350,2.13) | 4.51 (3.920,5.19) | 3.52 (3.13,3.96) |
| After | 1.61 (1.200,2.15) | 2.48 (1.990,3.08) | 2.09 (1.76,2.47) |
| Sp.-specific aEIRs | | | |
| An. funestus aEIR | 1.07 (0.983,1.17) | 1.24 (1.190,1.28) | 1.25 (1.20,1.29) |
| An. gambiae aEIR | 1.15 (1.080,1.22) | 1.08 (1.040,1.13) | 1.05 (1.02,1.08) |
| Age (years) | 1.05 (1.020,1.08) | 1.02 (0.999,1.03) | 1.02 (1.01,1.04) |
| During | 1.65 (1.310,2.07) | 3.80 (3.270,4.42) | 2.96 (2.62,3.35) |
| After | 1.68 (1.230,2.29) | 2.12 (1.700,2.63) | 1.79 (1.50,2.12) |
