# Supplementary material for: Disentangling the roles of different vector species during a malaria resurgence in Eastern Uganda
Source: PLOS Glob Public Health. 2025 Dec 11;5(12):e0004436. doi: 10.1371/journal.pgph.0004436 (PMC12697997; doi:10.1371/journal.pgph.0004436)
Supplement: S5 Table — aEIR estimated using modeled vector counts and household mean SR. All aEIRs are log2-transformed. (DOCX) [file pgph.0004436.s009.docx]

**S5 Table. Alternative aEIR: aHRs assuming a linear relationship between all covariates and the log hazard.**

aEIR estimated using modeled vector counts and household mean SR. All aEIRs are log_2_-transformed.

|  | Busia | Tororo | Overall |
| --- | --- | --- | --- |
| Total aEIR | | | |
| Total aEIR | 1.20 (1.140,1.25) | 1.19 (1.15,1.22) | 1.20 (1.17,1.23) |
| Age (years) | 1.04 (1.010,1.07) | 1.02 (1.00,1.04) | 1.02 (1.01,1.04) |
| During | 1.83 (1.440,2.32) | 4.56 (3.95,5.28) | 3.64 (3.22,4.11) |
| After | 1.75 (1.310,2.35) | 1.87 (1.53,2.29) | 1.86 (1.57,2.20) |
| Sp.-specific aEIRs | | | |
| An. funestus aEIR | 1.02 (0.956,1.09) | 1.14 (1.10,1.18) | 1.13 (1.10,1.17) |
| An. gambiae aEIR | 1.19 (1.130,1.25) | 1.13 (1.09,1.17) | 1.12 (1.09,1.16) |
| Age (years) | 1.04 (1.010,1.07) | 1.02 (1.01,1.04) | 1.03 (1.01,1.04) |
| During | 1.89 (1.490,2.40) | 4.50 (3.87,5.24) | 3.53 (3.11,4.00) |
| After | 1.85 (1.380,2.49) | 1.82 (1.48,2.23) | 1.78 (1.50,2.10) |
