# Supplementary material for: Disentangling the roles of different vector species during a malaria resurgence in Eastern Uganda
Source: PLOS Glob Public Health. 2025 Dec 11;5(12):e0004436. doi: 10.1371/journal.pgph.0004436 (PMC12697997; doi:10.1371/journal.pgph.0004436)
Supplement: Attachment — Submitted filename: reviewer_responses_IRB.docx [file pgph.0004436.s011.docx]

**Reviewer #1**

**1. I would have liked to know if the entomological data were analyzed by quarter or semi-annually, and if so, were the associations made even stronger? - because there seem to be a couple of peaks each year and maybe different depending on species.**

We agree with the reviewer that the seasonal peaks in mosquito populations are an important part of local vector ecology that may substantially influence the relationship between measures of mosquito exposure and *P. falciparum* infection risk. We want to clarify that, although we include entomological measures aggregated by time period (“before,” “during,” and “after” the malaria resurgence we describe) in Table 2 in order to summarize our data, the survival analysis itself did not use these aggregated measures. Instead, our primary analysis used daily estimates of annualized EIR, obtained by smoothing data from collections every two weeks.

**2. A major weakness of the assumptions is the collection of indoor host-seeking mosquitoes using light trap catches when IRS is the primary control tool and should be acting as a major repellent for those indoor catches in addition to the overall lethal effect, although they appropriately speak to this weakness in the discussion.**

We agree that this is an important weakness of the study (currently included, as the reviewer points out, in the discussion).

**3. Another weakness they don’t address that they only tested/account for P. falciparum, and only using an ELISA assay that can be less sensitive than molecular assays.**

The reviewer raises an important point, also mentioned by reviewer 2 (question 14). We have not used molecular assays for sporozoite identification in our cohorts, and acknowledge that the lower sensitivity of this test could result in underreported sporozoite rates. We have added the following caveat to the discussion:

*“Our use of an ELISA sporozoite assay that can be less sensitive than molecular assays, inability to quantify mosquito salivary gland sporozoite loads, and focus on P. falciparum to the exclusion of other malaria parasites also limited our ability to fully characterize risk associated with exposure to the two vector species.”*

**4. Lastly, the data are lumped together as FF+SS are both clothianidin-based, but infection incidence doesn’t seem to rise until the SS is implemented rather than FF, and the latter contains added pyrethoid and I’m sure the formulations/long-lasting efficacies are different – can this all be more solidly connected to the SS spray alone?**

We agree that the distinctions between the two formulations are potentially important, and do not mean to imply that they are interchangeable. We base our classification on prior work from our group (Kamya et al, 2024). There, the increase in malaria incidence in relation to the switch from Actellic to Fludora Fusion (i.e., before the introduction of Sumishield) is particularly evident in Fig. 2. Furthermore this paper also reported high tolerance of An. funestus to clothianidin, but full susceptibility to Actellic, In our analysis, the change may be less evident in part because of the truncated time period that we examine here – our first timepoint postdates the first round of Fludora Fusion, by which time there had already been an increase from a pre-Fludora Fusion baseline.

**Reviewer #2:**

**1. Figure 1 - no legend included? I understand it is a simple figure so may not need it; just want to verify it wasn’t missed.**

We have opted not to include description of this figure beyond that included in the title.

**2. Supplemental Figures S1 and S2 – would be helpful to include in the legend the description of the line in the plot. Presumably based on its color it represents the Busia trends of time to infection across individuals. I would want to see the same line for Turoro as well, to better depict comparisons you make in the text (specifically stating time to next infection is longer in Busia).**

This is a helpful point to clarify – what appears to be a trend line in this figure is a series of closely spaced individual boxplots. We have added a description in the legend of the relevant components of the boxplot (after Tukey): i.e., marks indicating the median and first and third quartiles, two whiskers, and outlying points with color varying by site.

*“The boxplot includes marks indicating the median and first and third quartiles, two whiskers, and outlying points, with color varying by site.”*

**3. Given the proximity of Busia households and the also those in the Turoro border region to Kenya (as evidenced by Figure 1), I think you should also include in the intro or discussion the basics about what is known about malaria cases in this Northwestern Kenya region during the same time frame and their infection control measures.**

We appreciate this important point. While we don’t have similarly detailed surveillance data from Busia County in Kenya, modeled annual incidence rates from the Malaria Atlas Project suggest that the county did not experience a similar rise in malaria incidence during the period covered in this study. Otherwise, vector bionomics are similar on the Kenyan side. PMI funded mass distribution of PBO ITNs in Busia in 2021, but large-scale IRS was not implemented during the study period. We have added these details to the methods section (under “Study Participation”).

*“In the adjoining Kenyan county of Busia, which we did not survey, modeled annual incidence rates from the Malaria Atlas Project suggest that there was no similar rise in malaria incidence during the period covered in this study [1]. The President’s Malaria Initiative funded mass distribution of pyrethroid piperonyl-butoxide ITNs in the county in 2021, but did not implement IRS during the study period.”*

**4. Not absolutely necessary, but I would consider the benefit of another component to Figure 1 being a longitudinal depiction of the study timeframe with different IRS insecticide and LLIN distribution interventions highlighted (e.g. arrows above the line for Turoro interventions, arrows below the timeline for Busia interventions)**

We agree that this would act as a helpful reference for the reader. We have included a timeline of relevant interventions as a new supplementary figure (see Fig. S1. Caption: “Timeline of recent indoor residual spraying (IRS) and long-lasting insecticidal net (LLIN) distribution campaigns by district, including names of insecticide formulations and LLIN models.”)

**5. Would appreciate better/more explicit description of malaria cases (rates of symptomatic vs asymptomatic) at least in the methods. What percentage of people at baseline enrolment time had asymptomatic parasitemia?**

We appreciate the opportunity to clarify this point – the formal analysis focuses on the incidence of infection (including asymptomatic and symptomatic cases), which we have clarified by adding the following to the methods section (under “Survival analysis”):

*“Incident P. falciparum infection was defined as the first occurrence of either positive qPCR or positive microscopy, regardless of the presence of symptoms, following either a prior documented malaria treatment course or a spontaneous clearance (i.e., asymptomatic individuals who clear parasitemia without treatment).”*

We also agree that describing the prevalence of parasitemia at baseline would provide important context. Because the study used dynamic enrollment, with “Tororo, away” added much later than Busia and Tororo near the border, we have avoided comparing across multiple time periods by restricting this analysis to January-August 2021, or the period of between the start of enrollment at Tororo away from the border and the start of the resurgence as designated in the text. Within this period, to provide a sense of baseline prevalence, we have calculated the percentage of individuals who were positive for *P. falciparum* by PCR at their first evaluation. We have also added the percentage of incident infections that were accompanied by fever at the time of diagnosis. We have added the following to the results section (under “Change in risk of infection over the study period”):

*“Between the start of enrollment at Tororo away from the border and start of the malaria resurgence as defined above (January-August 2021), the percentage of individuals who were positive for P. falciparum by PCR at the first evaluation falling within the analysis period was 42.1% (32/76) in Busia, 53.9% (69/128) in Tororo near the border, and 12.8% (15/117) in Tororo away from the border…*

*26.3% (163/620) of incident infections in our analysis were accompanied by fever in Busia, 28.8% (290/1006) in Tororo near the border, and 38.8% (357/921) in Tororo away from the border. ”*

**6. You refer to spontaneous clearances a lot without explicitly defining them as presumably untreated asymptomatic people who naturally clear – it would be better to state that (pg 8 line 151-152), as well as to clearly state that 3 consecutive negative screening periods would cover 3 months (based on q28 day sampling?) – which I agree is a reasonable time to assume that newly detected parasitemia is more likely from a new infection. Of course it is possible, as you allude to, that if a person had asymptomatic infection with no treatment required, their subsequent parasitemia or symptomatic case could be from prior parasite versus from new infection from new mosquito bite. I know that you also included data in your supplemental info about a more “conservative” analysis with incident infections only considered after treatment, presumably to account for this scenario. I think it would just overall help to be more clear about in your manuscript about these nuances, but why you would ultimately make the same conclusions – could even just add a sentence in the discussion.**

We appreciate the comment and agree that we can provide clearer justification of the methodologies used in both the primary and supplementary analyses (which are as the reviewer laid out in this comment). We have added further context for these decisions to the methods section under “Survival analysis”:

*“Incident P. falciparum infection was defined as the first occurrence of either positive qPCR or positive microscopy, regardless of the presence of symptoms, following either a prior documented malaria treatment course or a spontaneous clearance (i.e., asymptomatic individuals who clear parasitemia without treatment). Spontaneous clearances were defined as three negative qPCR results in succession (covering approximately 3 months, based on sampling every 28 days as previously described [2]). An alternate, more conservative analysis in which we only considered incident infections occurring after documented treatment (intended to address the possibility that persistent low-density parasitemia temporarily falling below the threshold of detection by qPCR could be falsely labeled as a clearance and subsequent reinfection) was also conducted and is reported as a supplementary result.”*

**6. I also think somewhere in your manuscript you should describe seasonality of malaria cases in this region. Were there any significant weather pattern or case seasonality changes during the resurgence (i.e. changes in rainfall) that should be considered – given An. funestus is slightly more adapted to dryer conditions than An. gambiae?**

The reviewer raises an important point – climatic (and, more broadly, environmental) changes are absolutely a plausible contributor to changing vector species composition. While a full analysis of environmental drivers is beyond the scope of this paper (which is not focused on formally analyzing the causes of the change in species composition we describe), our group has been conducting studies in Tororo/Busia districts for decades and there were no obvious changes in precipitation or temperature during the study period that would have favored An. funestus. We have added the following description of local climate to the methods section under “Study participation”:

*“The region has historically experienced two seasonal peaks in malaria transmission following the rainy seasons, with precipitation peaking in April and November (based on the 2000-2018 mean) [3].”*

**7. Similarly, for more context regarding regional intervention differences – why doesn’t Busia get have IRS implemented? Are there other significant differences in the Busia landscape? What happened with bednet distribution in 2023 (presumably every 3 year planned distribution schedule) – did this occur and could this have affected the subsequent case decline in Turoro again?**

The reviewer raises an important point of clarification. There were no significant baseline differences between Tororo and Busia in terms of environment or vector bionomics that led to IRS implementation in the former but not the latter. Due to limited resources, the National Malaria Control Program selects a small subset of districts for targeted IRS campaigns, and Busia’s omission was not based on any stated exclusion criteria.

As for ITN distribution campaigns, the reviewer is correct that ITNs were distributed every 3 years, with the most recent distributions occurring in 2023: August 2023 in Tororo (Yorkool and PermaNet 3.0) and November 2023 in Busia (Interceptor G2). We have added these dates to the methods section (under “Study Participation”) and the new supplementary figure described in our response to comment 4. The Tororo 2023 distribution date substantially postdates the initial drop in local malaria incidence, and we feel it is unlikely to have been a primary driver of changing malaria risk.

The corresponding section has been changed to:

*“Pyrethroid long-lasting insecticidal nets (LLINs) were distributed in 2013, 2017 and June 2020, and a mix of LLINs treated with deltamethrin (Yorkool) and deltamethrin plus piperonyl butoxide (PermaNet 3.0) in August 2023. IRS was conducted with the carbamate bendiocarb from 2014 to 2015, the organophosphate Actellic (pirimiphos-methyl) from 2017 to 2019, and with Fludora Fusion (clothianidin and deltamethrin) starting in 2020. During the study period, there were three additional rounds of IRS in Tororo: Fludora Fusion in March 2021, SumiShield (clothianidin) in March 2022, and a return to Actellic in March 2023, which was followed by a decline in malaria transmission [4]. In Busia, pyrethroid LLINs were distributed in 2013 and 2017, LLINs treated with deltamethrin plus piperonyl butoxide (PermaNet 3.0) in December 2020, and LLINs treated with alphacypermethrin and clorfenapyr (Interceptor G2) in November 2023, but no IRS was performed.”*

**8. Some clarifications regarding Table 2: I understand your data implies there was not a malaria resurgence in Busia, nor changes in vector species, over the period of interest. Still, comparing segmented Turoro data (divided as before/during/after IRS insecticide change) to Busia data collectively over that whole period makes it harder to verify. The Busia data for Table 2 would be helpful to see in a similar before/during/after breakdown (at least in supplemental info) for better comparison.**

We agree with the reviewer that it would be helpful to present the data from Busia and Tororo using the same temporal divisions. We have updated Table 2 accordingly.

**9. It was surprising and somewhat confusing to see aEIR of 0 at so many times – presumably driven by the SR with no infected mosquitoes collected for a given time. Normally would not expect the IQR to be 0 so often given the overall high malaria prevalence in this region? Please clarify your thoughts.**

The reviewer points out an important difficulty inherent to entomological surveillance at smaller scales – the modal sporozoite rate at the level of an individual night of collection is typically zero, reflecting the small sample sizes used for these calculations and the low probability that an individual mosquito will be found to carry sporozoites even in regions with high-intensity transmission. For comparison, Table 2 also shows species-specific annualized entomological inoculation rates calculated by aggregating vector counts and sporozoite rates over the full study period for each site, which are majority nonzero. We have added this caveat to the discussion:

*“More generally, entomological surveillance at small scales is invariably limited by noise and by the zero inflation that results from estimating sporozoite rates using small sample sizes. Although we attempted to address these issues through smoothing, this difficulty remains a fundamental issue for EIR estimation, particularly with the low nightly mosquito captures we described. Relatively small changes in vector density or sporozoite rates can translate to large changes in estimated EIR, meaning our results should be interpreted with caution. For instance, the changes in An. funestus aEIR shown in Table 2 are driven in part by a rise in An. funestus SR during the study period. While potential contributors include ecological factors such as increased feeding success or longevity in An. funestus or temporarily decreased rates of An. funestus emergence with a consequently aging, sporozoite-enriched population, this change could also reflect an artifact of the larger sample size available for sporozoite rate estimation during the resurgence.”*

**10. I would better define the numbers scale in the legend. Presumably number of collections equates to households? What are the An. gambiae or An. funestus counts – an actual number of mosquitoes collected? If so, this is quite low for mosquitoes collected in each house. It is confusing because, looking at the before/during/after of An. funestus count in Turoro near, there is a decline over all times (like An. gambiae, though much less drastic) – which seemingly doesn’t align as well the idea of with An. funestus influx driving a malaria resurgence. This would make it seem the SR changes (increases) for An. funestus are driving aEIR data. It’s more intuitive to think about mosquito numbers changing with insecticide use – but why would An. funestus SR rates change over the study period? This is another reason it would be helpful to see Busia data also divided in time for comparison.**

Taking the reviewer’s comments one by one:

-“Number of collections” refers to the number of nights of collection represented (which could include multiple nights at an individual household). We have clarified this by adding additional information to the table legend:

*“SR=sporozoite rate; aEIR=annualized entomological inoculation rate; No. collections = number of household-nights of mosquito collection.”*

-We agree that the number of mosquitoes collected per night is low, and this was originally reported in the parent study [4]. We have added additional mention of this as a caveat in the discussion:

*“Although we attempted to address these issues through smoothing, this difficulty remains a fundamental issue for EIR estimation, particularly with the low nightly mosquito captures we described.”*

-The reviewer correctly points out that the table does not present as clear as a picture of the entomological trends we describe as does Fig. 2. In particular, Tororo near the border shows absolute declines in the medians of all mosquito counts, although the median *An. funestus* vector count increases during the resurgence. The coarse time divisions we use in Table 2 (and which are used in our survival analysis as fixed effects but are not used to aggregate modeled aEIR) do not usefully summarize the seasonal variation that can be seen in the figure. Importantly, however, Table 2 still demonstrates changes in the dominant vector species during the resurgence, with *An. funestus* outnumbering the previously more common *An. gambiae*. This point also implies another critique, which is that we do not mean to imply that our data definitively demonstrate an “influx” of *An. funestus*, but rather a change in species composition. We have added a more nuanced description of these trends to the results section:

*“In Tororo near the border, median An. funestus aEIR rose from a baseline of 0 (IQR 0-7.3) to 8.9 (0-20.8) during the resurgence and returned to 0 (0-0) afterwards. Median nightly An. funestus counts remained stable during the resurgence (1.4 [0.9-2.3 ] before to 1.2 [0.6-2.2] during), while SR rose (0% [0-1] before to 1.7% [0-3.1] during). An. gambiae aEIR fell from 14 (0-27.5) to 7.8 (0-11.1) to 0 (0-0) over the same time periods.*

*In Tororo away from the border, median An. funestus aEIR rose from 0 (0-0) to 4.5 (0-11.2) during the resurgence and returned to 0 (0-0) afterwards. Median nightly An. funestus counts rose during the resurgence (0.1 [0.1-0.2] before to 0.6 [0.2-1.1] during), as did SR (0 [0-0] before to 1.5 [0-2.5] during).”*

We have also clarified this nuance in the discussion:

*“In Tororo, during a period after a change in IRS insecticide when malaria incidence rose and An. funestus became the dominant local malaria vector [4], increased household-level An. funestus EIR was a stronger predictor of changes in P. falciparum infection rates than An. gambiae EIR as measured using CDC light trap collections.”*

-Finally, we agree with the reviewer that the increase in *An. funestus* SR during the study period requires more explanation. Potential contributors include increased feeding success or longevity in *An. funestus*, temporarily decreased rates of *An. funestus* emergence with a consequently aging, sporozoite-enriched population, an artifact of the larger number of collection household-nights (and therefore fewer zeroes in sporozoite rate estimation) during the resurgence, or a combination of these factors. Along with additional description of the SR dynamics in the results section (shown above), we have added the following to the discussion:

*“For instance, the changes in An. funestus aEIR shown in Table 2 are driven in part by a rise in An. funestus SR during the study period. While potential contributors include ecological factors such as increased feeding success or longevity in An. funestus or temporarily decreased rates of An. funestus emergence with a consequently aging, sporozoite-enriched population, this change could also reflect an artifact of the larger sample size available for sporozoite rate estimation during the resurgence.”*

**11. This previous point brings up the general issue of how the relatively low overall number of sporozoite-infected mosquitoes detected makes it difficult to precisely evaluate temporal trends and make significant conclusions. I appreciate that your modeling work tries to overcome this limitation – but I think it is worth mentioning in your discussion/limitations. (Especially also considering we don’t have any data about quantitative sporozoite burden between An. gambiae and An. funestus here, another factor which can contribute to subsequent infection risk – though I recognize it’s not realistic to assess sporozoite loads with your sample set.)**

We agree with this important point - see responses to comments #9 and #10, as well as the following caveat we have added to the discussion about our sporozoite assay:

*“Our use of an ELISA sporozoite assay that can be less sensitive than molecular assays, inability to quantify mosquito salivary gland sporozoite loads, and focus on P. falciparum to the exclusion of other malaria parasites also limited our ability to fully characterize risk associated with exposure to the two vector species.”*

**12. Likewise, regarding data in Fig 2 – An. gambiae and An. funestus data look quite similar regarding vector numbers in Turoro areas for much of the main period of interest as do the species-specific SR data especially for Turoro near and Busia. And yet aEIR increases more notably for An. funestus. Hard to see clear pattern in graphed mosquito capture and SR data, but the modeling implies the aEIR changes are bigger. I think this requires more nuanced interpretation of results – making clear that your conclusions are based on aEIR estimates and modeling and noting small differences in vector and SR detection can show up bigger in these EIR-based models, though obviously the infection rise over this period is striking.**

We agree with this point, which goes along with the concerns about sporozoite rate measurement noted above – EIR-based results, and particularly modeled EIRs, should be interpreted with caution. We have added the following to the discussion:

*“More generally, entomological surveillance at small scales is invariably limited by noise and by the zero inflation that results from estimating sporozoite rates using small sample sizes. Although we attempted to address these issues through smoothing, this difficulty remains a fundamental issue for EIR estimation, particularly with the low nightly mosquito captures we described. Relatively small changes in vector density or sporozoite rates can translate to large changes in estimated EIR, meaning our results should be interpreted with caution. For instance, the changes in An. funestus aEIR shown in Table 2 are driven in part by a rise in An. funestus SR during the study period. While potential contributors include ecological factors such as increased feeding success or longevity in An. funestus or temporarily decreased rates of An. funestus emergence with a consequently aging, sporozoite-enriched population, this change could also reflect an artifact of the larger sample size available for sporozoite rate estimation during the resurgence.”*

**13. Also, following the data from Figure 2 input to generate the aHR data in Table 3 – it is important to contextualize and note that despite the story and data fitting nicely regarding the switch of IRS insecticide, rise in malaria cases, and noted relative increase in An. funestus mosquito presence, the differences in species-specific aEIR-based aHR calculations between Busia and Turoro are overall still small. Thus, I do especially appreciate the discussion acknowledgement about EIR measures being complex and hard, particularly with An. gambiae having 2 subspecies, and different behavioral characteristics of the various Anopheles vectors and how this could skew the modeling here based on your indoor capture data. It will be interesting to see the data from the publication you mentioned that is under review regarding outdoor exposures. (Nothing requested to change, just a comment.)**

We appreciate this comment – the paper we mention here is now published, and the citation has been updated.

**14. Did you consider using PCR as the method for sporozoite identification rather than ELISA? Would be interesting to have simultaneously looked at presence of different malaria species in your cohort (given some others are finding potential associations between An. funestus and P. ovale, for example (https://www.medrxiv.org/content/10.1101/2024.10.09.24315124v1)).**

This is a helpful question that was also raised by reviewer 1 (question 3). We have not used molecular assays for sporozoite identification in our cohorts, and have added the following caveat to the discussion:

*“Our use of an ELISA sporozoite assay that can be less sensitive than molecular assays, inability to quantify mosquito salivary gland sporozoite loads, and focus on P. falciparum to the exclusion of other malaria parasites also limited our ability to fully characterize risk associated with exposure to the two vector species.”*

1.	Hay SI, Snow RW. The Malaria Atlas Project: developing global maps of malaria risk. PLOS Med. 2006;3(12):e473.

2.	Briggs J, Teyssier N, Nankabirwa JI, Rek J, Jagannathan P, Arinaitwe E, et al. Sex-based differences in clearance of chronic Plasmodium falciparum infection. Elife. 2020;9:e59872.

3.	Climate Hazards Center Infrared Precipitation with Stations version 3. CHIRPS3 Data Repository <https://doi.org/10.15780/G2JQ0P>. 2025.

4.	Kamya MR, Nankabirwa JI, Arinaitwe E, Rek J, Zedi M, Maiteki-Sebuguzi C, et al. Dramatic resurgence of malaria after 7 years of intensive vector control interventions in Eastern Uganda. PLOS Glob Public Health. 2024;4(8):e0003254.
